# Xylem Hydraulics of Two Temperate Tree Species with Contrasting Growth Rates

**DOI:** 10.3390/plants13243575

**Published:** 2024-12-21

**Authors:** Ai-Ying Wang, Yi-Jun Lu, Han-Xiao Cui, Shen-Si Liu, Si-Qi Li, Guang-You Hao

**Affiliations:** 1 School of Life Sciences and Engineering, Shenyang University, Shenyang 110044, China; aiyingwqepq@163.com (A.-Y.W.); luyijun0306@163.com (Y.-J.L.); lsq18183756497@163.com (S.-Q.L.); 2 Liaoning Key Laboratory of Urban Integrated Pest Management and Ecological Security, College of Life Science and Bioengineering, Shenyang University, Shenyang 110044, China; 3 CAS Key Laboratory of Forest Ecology and Management, Institute of Applied Ecology, Chinese Academy of Sciences, Shenyang 110016, China; 15666853516@163.com (H.-X.C.); liushensi19@mails.ucas.ac.cn (S.-S.L.); 4 University of Chinese Academy of Sciences, Beijing 100049, China

**Keywords:** cavitation resistance, plant water-use strategy, radial growth rate, wood anatomical, xylem hydraulics

## Abstract

Hydraulic functionality is crucial for tree productivity and stress tolerance. According to the theory of the fast–slow economics spectrum, the adaptive strategies of different tree species diverge along a spectrum defined by coordination and trade-offs of a suite of functional traits. The fast- and slow-growing species are expected to differ in hydraulic efficiency and safety; however, there is still a lack of investigation on the mechanistic association between tree growth rate and tree hydraulic functionality. Here, in a common garden condition, we measured radial growth rate and hydraulic traits in a fast-growing (*Populus alba* L. × *P. berolinensis* Dippel) and a slow-growing tree species (*Acer truncatum* Bunge), which are both important tree species for afforestation in northern China. In line with the contrasts in radial growth rate and wood anatomical traits at both the tissue and pit levels between the two species, stem hydraulic conductivity of the *Populus* species was significantly higher than that of the *Acer* species, but the resistance to drought-induced xylem cavitation was the opposite. A trade-off between hydraulic efficiency and safety was observed across the sampled trees of the two species. Higher water-transport efficiency supports the greater leaf net photosynthetic carbon assimilation capacity of the *Populus* species and hence facilitates fast growth, while the conservative hydraulic traits of the *Acer* species result in a slower growth rate but enhanced drought tolerance.

## 1. Introduction

In recent years, climate change has resulted in a rising frequency of extreme droughts and heatwaves, contributing to increased tree mortality. This trend poses a significant threat to forest health, particularly in regions where water resources are scarce [1,2]. Drought is recognized as an important factor limiting tree growth, and hydraulic dysfunction is a key mechanism contributing to tree mortality. Understanding the effects of hydraulic dysfunction on tree mortality is important for effective management of forest ecology and for improving ecosystem resilience under conditions of increased drought [3,4]. Plant hydraulic architecture refers to the different structures and functions related to water conduction formed by a plant species under specific environmental conditions [5]. Tree species show significant differences in hydraulic failure risk due to their unique hydraulic characteristics and water-use strategies. Even in the same region, different tree species facing the same drought stress can have different adaptive strategies [6]. They may also have different sensitivities to drought caused by climate change [7]. Thus, investigating the differences in hydraulic architecture of different tree species is important for addressing the problem of tree decline and mortality caused by drought in the background of climate change. Especially in areas where water resources are limited, these studies can provide a scientific basis for the selection of suitable tree species for afforestation.

Significant differences in hydraulic architecture exist between plants, which fundamentally affect their water-transport efficiency and growth performance. Research indicates that fast-growing tree species generally have higher hydraulic conductivity, larger ratios of leaf and branch xylem cross-sectional areas, and lower resistance to embolism [8,9]. Hydraulic conductivity is a measure that reflects the efficiency of water transport in plant xylem, indicating how permeable the sapwood is to water. Hydraulic conductivity is influenced by structural features, including the diameter of conduits or tracheids within the sapwood. The diameter of xylem conduits is positively associated with water-transport efficiency with larger conduits facilitate faster and more effective water movement. However, this increase in diameter can also compromise the xylem’s ability to withstand drought-induced embolism [10]. Water transport through a conduit is proportional to the fourth power of its diameter, which implies that a larger diameter results in significantly greater water-transport efficiency [11]. Furthermore, studies have revealed a positive correlation between xylem water conductance and stomatal conductance, facilitating a balance between water supply and demand during plant transpiration [12,13]. Xylem hydraulics and leaf photosynthetic gas exchange exhibit a synergistic relationship [14]. A robust positive correlation has been observed across various species between stem hydraulic conductivity and the capacity of leaves to assimilate carbon during photosynthesis [12,15,16]. There may be significant differences in water physiological mechanisms and photosynthetic carbon assimilation between fast-growing and slow-growing tree species. Conducting comparative studies on water transport and the associated carbon physiology between fast-growing and slow-growing tree species is of great significance for understanding the differences in their growth strategies and adaptability to environmental water conditions. Considerable variations in the anatomical structure of xylem can be observed among various tree species. These structural differences form a crucial trade-off between hydraulic safety and hydraulic efficiency among the species [16]. In addition to the anatomical features at the level of xylem tissue, the pit structure characteristics of vessels or tracheids also have a significant impact on tree water-transport efficiency and hydraulic safety [17]. Research has demonstrated that pit structures within the xylem play a pivotal role in plant water transport, with more than 50% of the overall hydraulic resistance originating from these structures [18].

The higher permeability of the pit membrane can effectively improve the water-transfer efficiency between conduits, but it also raises the risk of embolism spreading through air seeding [19]. Studies have shown that the size and thickness of the pores in porous pit membranes are important indicators that affect the xylem resistance to embolism [19,20]. For example, the pore size of the pit membrane is influenced by the thickness of the membrane, which in turn affects the propagation of bubbles between adjacent conduits through the pores. Thick, porous membranes are less prone to stretching and therefore have stronger hydraulic safety [21,22]. It has also been found that the smaller the pit aperture, the greater the tension is needed for air bubbles to break through, and thus the stronger the resistance to xylem embolism [23]. Large-diameter conduits that typically have high hydraulic conductivity also have larger inter-conduit pits, resulting in a larger pit membrane surface area [18,19,24]. According to the air seeding hypothesis, the larger the micropores in the porous membrane, the easier it is for bubbles to propagate between adjacent conduits, thereby affecting the resistance to cavitation [19,21]. It is of great significance to investigate the differences in the pit-level structural characteristics of xylem between fast-growing and slow-growing tree species, in order to understand whether there is a trade-off between growth rate and drought resistance.

**Figure A1:**
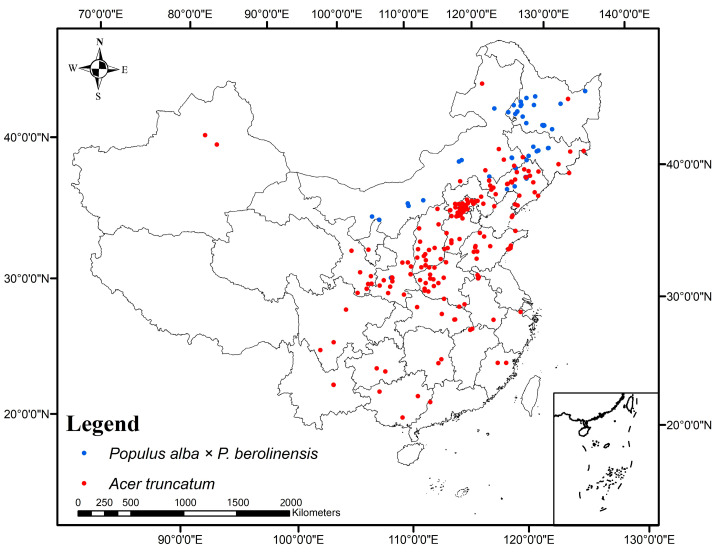
The occurrence records of *Populus alba* × *P. berolinensis* (*Pa.Pb*) and *Acer truncatum* (*At*) in China. The *Pa.Pb* data were obtained from the Literature Database (CNKI, https://www.cnki.net/, accessed on 13 December 2024) with 64 records collected, and the *At* data were obtained from the Global Biodiversity Information Facility (GBIF, https://www.gbif.org/, accessed on 13 December 2024) with 202 records found.

*Populus* species have been widely used for constructing windbreaks and shelter forests in northern China, particularly in the Three-North (Northwest, North, and Northeast) regions of China [25]. However, due to the problem of water-resource limitation, *Populus* plantations and windbreaks have experienced significant decline and mortality in recent years [26]. In contrast, the slow-growing native tree species, such as *Acer truncatum* Bunge, usually have much stronger drought resistance. It is of great importance to conduct comparative studies between the fast-growing but less drought-resistant *Populus* species and the slow-growing potential alternative tree species for afforestation in more drought-stressed environments, in order to better understand their respective advantages and disadvantages in afforestation usages. *Populus alba* L. × *P. berolinensis* Dippel is one of the commonly planted fast-growing hybrid poplars in northern China and *Acer truncatum* is a native tree species in China (Figure A1), which can potentially be a good alternative tree species for afforestation in areas that are too dry for *Populus* trees [27]. In the current context of increasingly frequent climate extremes, studying the hydraulic architecture and related functional traits of these two important tree species with distinct growth performance in northern China can inform afforestation in this area. This study explores the differences in xylem water conduction, xylem anatomy, and leaf gas exchange parameters between the two tree species. The study aimed to reveal differences in these functional traits between the two species and the relationships between them. The objective of this research was to investigate the physiological mechanisms underlying the contrasts in growth rate and stress tolerance between these two tree species. Based on this, the following main scientific hypotheses are proposed: (1) Compared with the *Acer* species, the *Populus* species has higher water-transport efficiency, but lower stress resistance; (2) Compared with *Acer truncatum*, the high water-transport efficiency of the *Populus* species supports its high leaf photosynthetic carbon assimilation capacity; (3) The poplar species’ “fast” growth strategy is facilitated by its high water-transport efficiency and elevated leaf photosynthetic carbon assimilation rate. In contrast, the “slow” growth strategy of *Acer truncatum* is supported by conservative traits, including higher wood construction costs that enhance hydraulic safety.

## 2. Results

### 2.1. Radial Growth Rate

**Figure 1:**
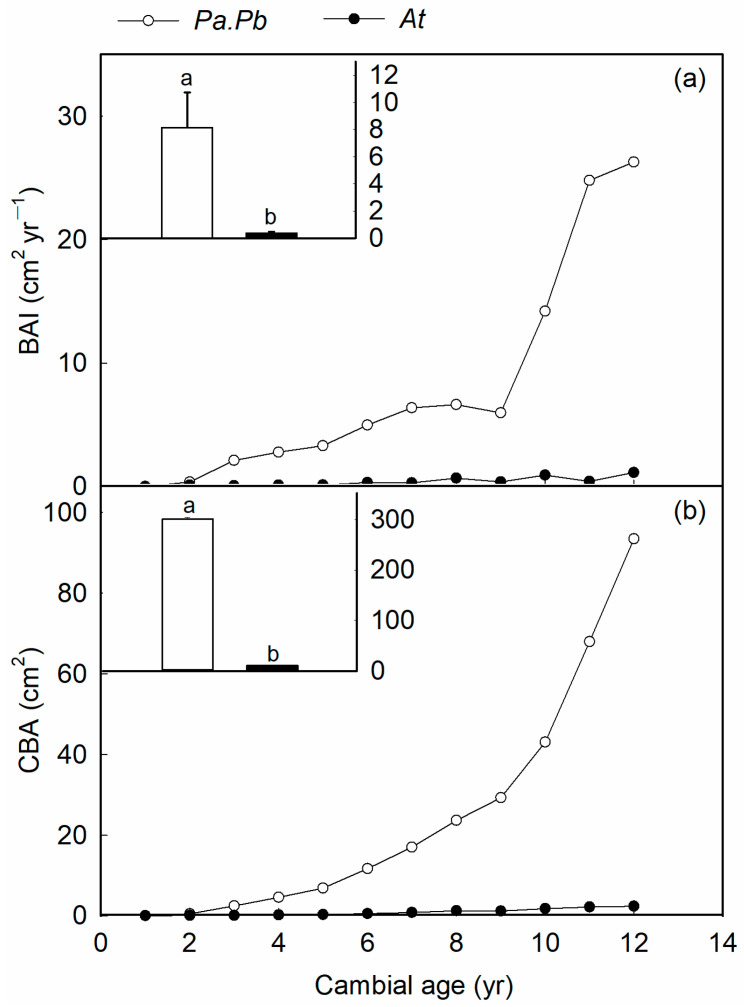
Comparison of radial growth of *Populus alba* L. × *P. berolinensis* Dippel (*Pa.Pb*) and *Acer truncatum* (*At*). (**a**) The basal area increment (BAI, cm^2^ yr^−1^) from cambial ages 1–12 years; (**b**) the cumulative basal area (CBA, cm^2^) at a cambial age of 12 years for each species (*p* < 0.01, *t* test). The BAI and CBA are mean values calculated from treecore analyses of all sampled trees (*n* ≥ 15). Each circle or dot represents the mean of four replicates of *Pa.Pb* and *At*, respectively. In both panels, different lowercase on top of the bars represent significant differences between the two species.

In the common garden growth environment, there is a significant difference in the radial growth rate between the two studied tree species. Before the cambium age of 12 years, both the basal area increment and the cumulative basal area of the two tree species showed a non-linear increase with age. The BAI at 12 years of cambium age of the *Populus* species is more than 36 times that of the *Acer* species, and the CBA was more than 29 times that of *Acer truncatum*. The average BAI and CBA of the *Populus* species were 8.15 cm^2^ yr^−1^ and 300.81 cm^2^, while the average BAI and CBA of *Acer truncatum* were 0.38 cm^2^ yr^−1^ and 11.07 cm^2^ (Figure 1). There were significant differences in BAI and CBA between the two species (*p* < 0.01 and *p* < 0.01).

### 2.2. Efficiency and Safety of Water Transport in Xylem

**Figure 2:**
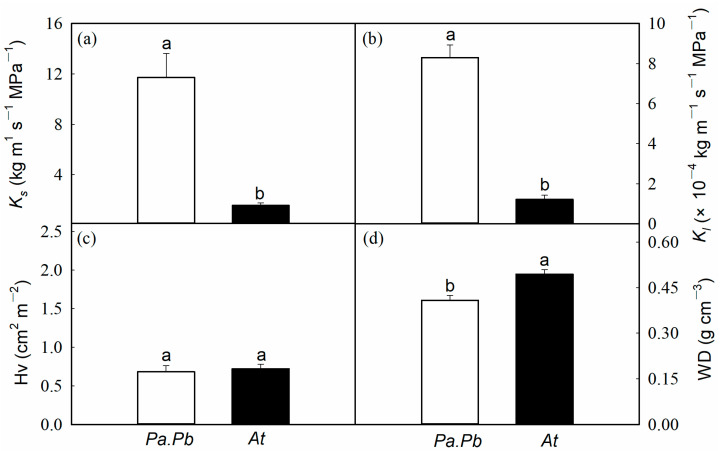
Comparisons of (**a**) sapwood hydraulic conductivity (*K*_s_), (**b**) leaf-specific conductivity (*K*_l_), (**c**) Huber value (Hv), and (**d**) wood density (WD) between *Populus alba* × *P. berolinensis* (*Pa.Pb*) and *Acer truncatum* (*At*). Error bars show 1 SE (*n* = 6). In each panel, different letters on top of the bars represent significant differences between the two species (*t* test, *p* < 0.05).

**Figure A2:**
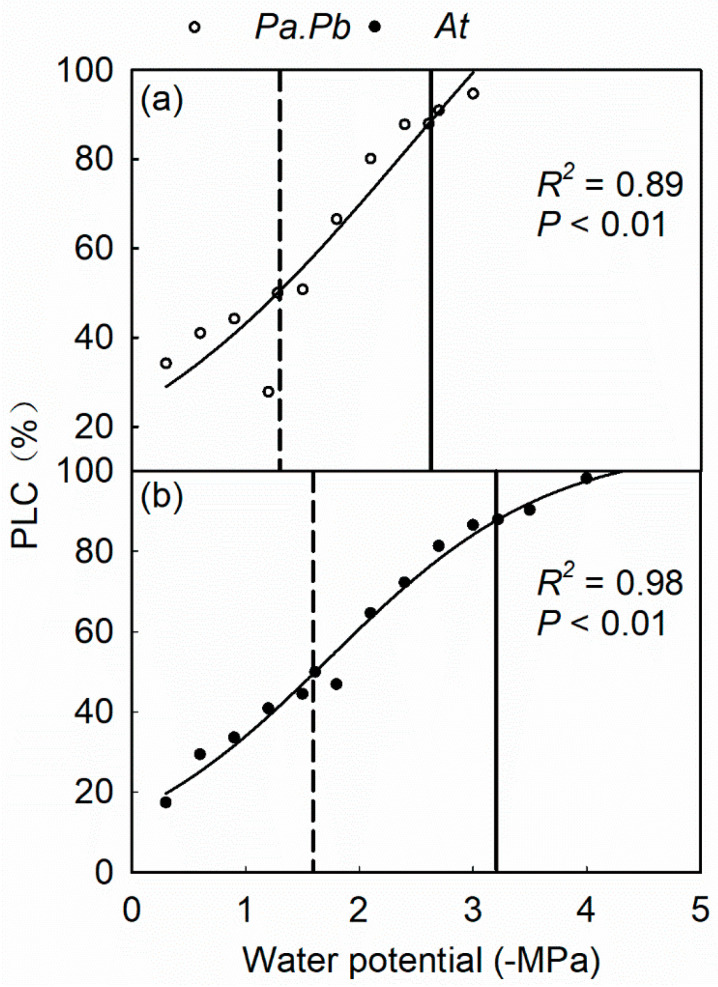
Stem vulnerability curves for (**a**) *Populus alba* × *P. berolinensis* (*Pa.Pb*) and (**b**) *Acer truncatum* (*At*) constructed using the centrifugal force method. All data points show mean values ± 1 SE (*n* = 4). Data were fitted using sigmoidal models. Dashed and solid vertical lines in each panel indicate xylem water potentials corresponding to 50 and 88% loss of hydraulic conductivity, respectively. PLC, changes in the percentage loss of conductivity. Each circle or dot represents the mean of four replicates of *Pa.Pb* and *At*, respectively.

There were significant differences in the hydraulic characteristics between the two species, with the *Populus* species having higher water-transport efficiency and the *Acer* species having higher water-transport safety. Compared with *Acer truncatum*, the *Populus* species showed significantly higher *K*_s_ and *K*_l_ (Figure 2; *p* < 0.01). The *K*_s_ and *K*_l_ of the *Populus* species were 11.72 kg m^−1^ s^−1^ MPa^−1^ and 8.3 × 10^−4^ kg m^−1^ s^−1^ MPa^−1^, respectively, while those of *Acer truncatum* were only 1.56 kg m^−1^ MPa^−1^ and 1.22 × 10^−4^ kg m^−1^ MPa^−1^, respectively, which were 7.5 and 6.8 times higher than those of *Acer truncatum* (Figure 2a,b; *p* < 0.01). There was no significant difference in Hv between the *Populus* and the *Acer* species, which were 1.60 and 0.76 cm^2^ m^−2^, respectively. The wood density of *Acer truncatum* was significantly higher than that of the *Populus* species, with values of 0.50 and 0.41 g cm^−3^, respectively (Figure 2d; *p* < 0.01). The hydraulic vulnerability curves of the branches indicated a significant difference in the ability of the two tree species to resist xylem embolism caused by drought (Figure A2). The *P*_50_ values of the *Populus* species and the *Acer* species were −1.26 MPa and −1.61 MPa (Table 1; *p* = 0.179), and the *P*_88_ values were −2.49 MPa and −3.12 MPa, respectively (Table 1; *p* < 0.01). The hydraulic safety margin calculated based on the difference between midday water potential and *P*_50_/*P*_88_ showed significant differences between the two tree species, with the stem hydraulic safety margin of *Acer truncatum* being significantly larger than that of the *Populus* species (Table 1; *p* < 0.05). The HSM_50_ and HSM_88_ of the *Acer* species were both positive values (0.31 MPa and 1.82 MPa, respectively), while those of the *Populus* species were both negative values (−0.98 MPa and −1.26 MPa, respectively).

### 2.3. Characteristics of Xylem Structure

**Figure 3:**
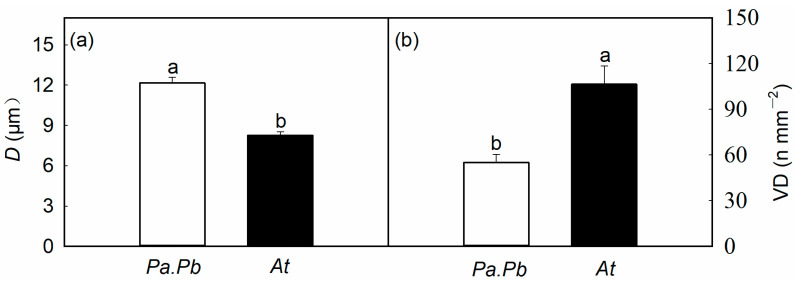
Comparisons of (**a**) vessel diameter (*D*), (**b**) vessel density (VD) between *Populus alba* × *P. berolinensis* (*Pa.Pb*) and *Acer truncatum* (*At*). Error bars show 1 SE (*n* = 6). In each panel, different letters on top of the bars represent significant differences between the two species (*t* test, *p* < 0.01).

**Figure 4:**
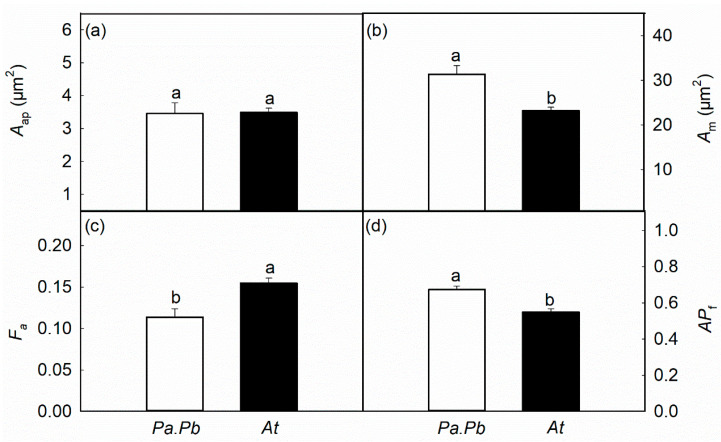
Comparisons of (**a**) the pit aperture area (*A*_ap_), (**b**) the pit membrane area (*A*_m_), (**c**) the aperture fraction (*F*_a_), and (**d**) the pit aperture shape index (*AP*_f_) between *Populus alba* × *P. berolinensis* (*Pa.Pb*) and *Acer truncatum* (*At*). Error bars show 1 SE (*n* = 6). In each panel, different letters on top of the bars represent significant differences between the two species (*t* test, *p* < 0.05).

Consistent with the differences in hydraulic properties, there were significant differences in the anatomical characteristics of the two species. The average diameter of the vessels in the *Populus* species was 12.16 μm, which was significantly larger than that in the *Acer* species (8.27 μm; Figure 3a; *p* < 0.01). The vessel density of the *Populus* species was 55.12 n mm^−2^, and the vessel density of *Acer truncatum* was 104.16 n mm^−2^ (Figure 3b; *p* < 0.01). Similarly, there were significant differences in xylem anatomical features of the two species at the pit level. The pit membrane area of the *Populus* species was 31.36 μm^2^, while that of *Acer truncatum* was 23.22 μm^2^ (Figure 4b; *p* < 0.01). The pit aperture fraction (*F*_a_) and the pit aperture shape index (*AP*_f_) of the *Populus* species were 0.11 and 0.67, respectively, while those in the *Acer truncatum* were 0.15 and 0.54, respectively (Figure 4c,d; *p* < 0.05).

### 2.4. Leaf Functional Traits

**Figure 5:**
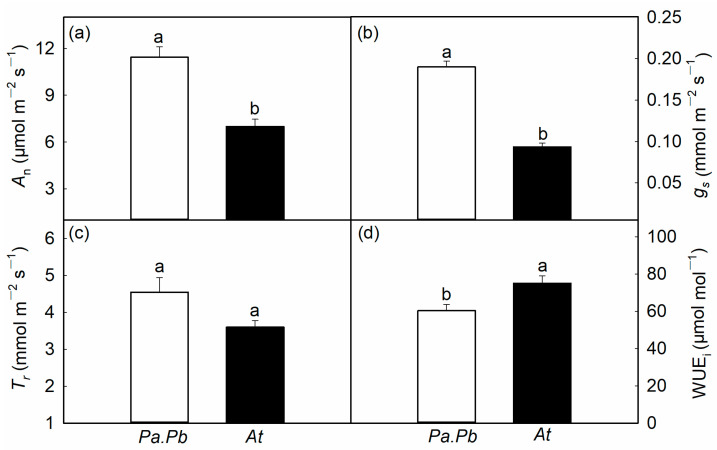
Leaf photosynthetic traits of *Populus alba* × *P. berolinensis* (*Pa.Pb*) and *Acer truncatum* (*At*). (**a**) Net photosynthetic assimilation rate (*A*_n_); (**b**) stomatal conductance (*g*_s_); (**c**) sap flow transpiration rate (*T*_r_); (**d**) water-use efficiency (WUE_i_). Error bars show 1 SE (*n* = 6). In each panel, different letters on top of the bars represent significant differences between the two species (*t* test, *p* < 0.05).

Compared to *Acer truncatum*, the *Populus* species exhibited a higher leaf gas exchange rate. The maximum net photosynthetic rate of *Acer truncatum* was 7.0 μmol m^−2^ s^−1^, while that of the *Populus* species was 11.4 μmol m^−2^ s^−1^ (Figure 5a; *p* < 0.01). The maximum stomatal conductance of *Acer truncatum* was 0.09 mmol m^−2^ s^−1^, significantly lower than that of the *Populus* species, which was 0.19 mmol m^−2^ s^−1^ (Figure 5b; *p* < 0.01). The photosynthetic intrinsic water-use efficiency of the *Populus* species and the *Acer* species showed a significant difference, with values of 60.3 and 75.03 μmol mol^−1^, respectively (Figure 5d; *p* < 0.05). There were significant differences in leaf morphology and anatomical traits between the two tree species (Table 2). The leaf area of the *Populus* species was 10.6 cm^2^, while that of *Acer truncatum* was 29.2 cm^2^. The *Populus* species was significantly lower than *Acer truncatum* (*p* < 0.05), while the LMAs of the *Populus* and the *Acer* species were 108.58 and 48.23 g m^−2^, respectively (Table 2; *p* < 0.05). The leaf thickness, palisade tissue thickness, sponge tissue thickness, and palisade to sponge tissue ratio of the *Populus* species were significantly higher than those of *Acer truncatum* (Table 2; *p* < 0.05).

### 2.5. Relationship Between Functional Traits

**Figure 6:**
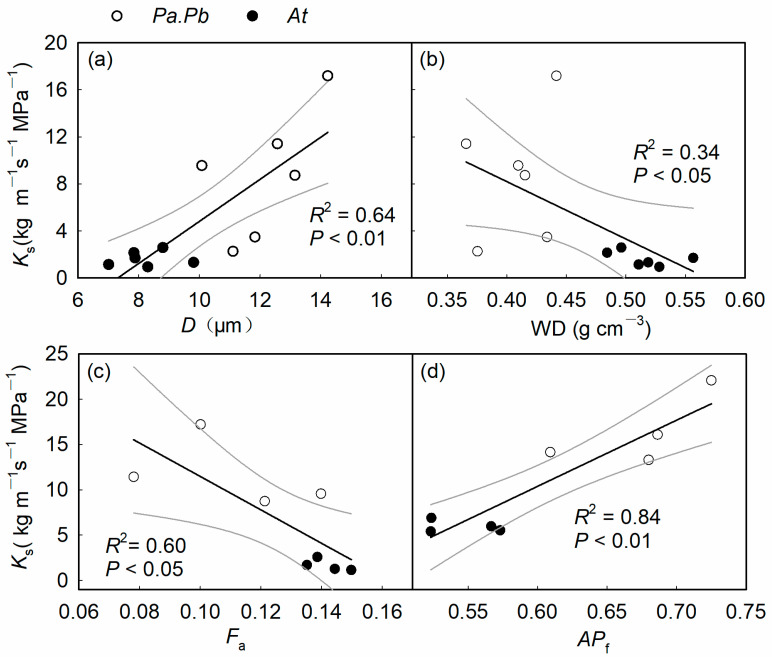
The sapwood hydraulic conductivity (*K*_s_) in relation to (**a**,**b**) xylem anatomical characters vessel diameter (*D*) and wood density (WD), (**c**,**d**) the pit anatomical characters of the aperture fraction (*F*_a_) and the pit aperture shape index (*AP*_f_). Each of the circles or dots represents an individual tree of *Populus alba* × *P. berolinensis* (*Pa.Pb*) and *Acer truncatum* (*At*), respectively.

**Figure 7:**
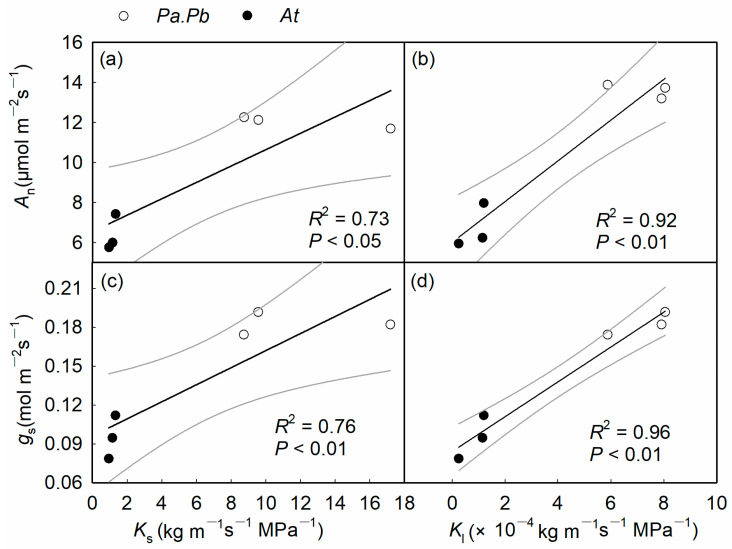
The sapwood hydraulic conductivity (*K*_s_) and leaf-specific conductivity (*K*_l_) in relation to the leaf photosynthetic traits (**a**,**b**) net photosynthetic assimilation rate (*A*_n_), (**c**,**d**) stomatal conductance (*g*_s_). Each of the circles or dots represents an individual tree of *Populus alba* × *P. berolinensis* (*Pa.Pb*) and *Acer truncatum* (*At*), respectively.

**Figure 8:**
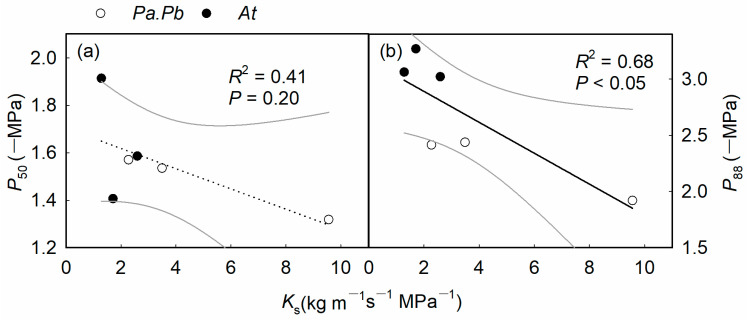
The sapwood hydraulic conductivity (*K*_s_) in relation to cavitation resistance. (**a**) Xylem water potentials corresponding to 50% loss of hydraulic conductivity (*P*_50_), (**b**) xylem water potentials corresponding to 88% loss of hydraulic conductivity (*P*_88_). Each of the circles or dots represents an individual tree of *Populus alba* × *P. berolinensis* (*Pa.Pb*) and *Acer truncatum* (*At*), respectively.

The stem hydraulic conductivity was closely related to the xylem anatomical traits at both the tissue and pit levels, as shown by a significant positive correlation between *K*_s_ and *D* (Figure 6a; *p* < 0.01), while *K*_s_ showed significant negative correlation with WD (Figure 6b; *p* < 0.05). At the pit level, *F*_a_, showed a significant negative correlation with *K*_s_ (Figure 6c; *p* < 0.05), while *AP*_f_ showed a strong positive correlation with *K*_s_ (Figure 6d; *p* < 0.01). There was a significant synergistic relationship between the hydraulic conductivity of the stem xylem and leaf gas exchange, manifested by significant positive correlations between *K*_s_ and *K*_l_ and *A*_n_ and *g*_s_ (Figure 7a–d; *p* < 0.05). In addition, there was a trade-off between hydraulic efficiency and safety indicators, with *K*_s_ showing marginal or significant negative correlations with *P*_50_ and *P*_88_ (Figure 8; *p* = 0.20, *p* < 0.05).

### 2.6. Results of the Principal Component Analysis

**Figure 9:**
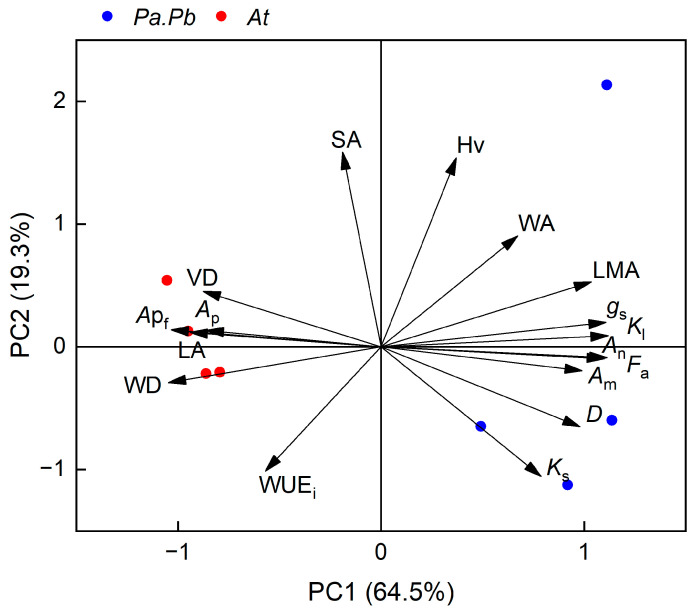
Results of the principal component analysis (PCA) of all the different species using 17 measured functional traits. Each of the red or blue dots represents an individual tree of *Populus alba* × *P. berolinensis* (*Pa.Pb*) and *Acer truncatum* (*At*), respectively. LA, leaf area; LMA, leaf mass per area; WD, wood density; WA, total wood area; SA, sapwood area; *K*_s_, sapwood hydraulic conductivity; *K*_l_, leaf-specific conductivity; Hv, Huber value; *D*, vessel diameter; VD, vessel density; *A*_n_, leaf net photosynthetic assimilation rate; *g*_s_, stomatal conductance; WUE_i_, instantaneous water-use efficiency; *A*_ap_, pit aperture area; *A*_m_, pit membrane area; *F*_a_, aperture fraction; *AP*_f_, pit aperture shape index.

The results of principal component analysis showed that the explanatory power of PC axis 1 and axis 2 were 64.5% and 19.3%, respectively (Figure 9). The functional traits related to resource acquisition, such as *D*, *g*_s_, LMA, WA, *A*_n_, *A*_m_, Hv, *K*_s_, *K*_l_, etc., were distributed on the positive half axis of PC1. The traits related to conservative resource use strategies, such as WD, VD, WUE_i_, LA, SA, *A*_ap_, *AP*_f_, etc., were distributed on the negative half axis of PC1. In the PCA plot, the two tree species were clearly separated by the PC1. The fast-growing *Populus* trees were distributed on the positive half axis of PC1, while the slow-growing *Acer* trees were distributed on the negative half axis of PC1.

## 3. Discussion

Our results indicated significant differences in xylem hydraulics and the associated carbon assimilation between the two studied species with contrasting growth rates in the common garden environment. *Populus* has significantly higher hydraulic conductivity, while *Acer* shows higher drought resistance and is better able to withstand xylem embolism under drought conditions. In addition, hydraulic conductivity, stomatal conductance, and leaf net photosynthetic rate were significantly higher in *Populus* than in *Acer*, implying that *Populus* has an advantage in photosynthesis and water-use efficiency. The differences in growth and water-transport characteristics between these two trees were closely related to their different resource utilization and drought-resistant strategies, reflecting their respective physiological and ecological characteristics for environment adaptation. Specifically, the poplar adopts an acquisitive strategy and has a high growth rate, which may be due to its high hydraulic conductivity and carbon assimilation capacity; however, *Acer truncatum* adopts a relatively conservative strategy, with a low growth rate but exhibiting strong resistance to drought.

### 3.1. Coordination Between Xylem Water Transport and Carbon Assimilation

The hydraulic efficiency and leaf gas exchange were positively correlated for both species, implying a close link between hydraulic efficiency and photosynthetic capacity [14]. Tree species with larger diameters of xylem conduits face less side wall resistance in water transport, which enables them to have higher water-transport efficiency [28]. High hydraulic conductivity allows trees to choose prodigal water-use strategies [8]. Additionally, a greater gas exchange rate also requires high water-transport efficiency to support, and high hydraulic conductivity enables plants to quickly replenish the water lost by transpiration from leaves, thereby maintaining a high photosynthetic carbon assimilation capacity [29], thereby endowing plants with high growth potential [8]. The *Populus* species achieved high hydraulic efficiency through its larger xylem conduits that supported more leaves for carbon assimilation [28,30], providing a fundamental basis for its high growth rate [31].

### 3.2. The Trade-Off Between Hydraulic Efficiency and Safety

The disparities in hydraulic properties between the two species illustrate a trade-off between water-transport efficiency and hydraulic safety [32]. Notably, *Acer truncatum* possesses a significantly smaller vessel diameter compared to the *Populus* species. This ensures the hydraulic safety of the xylem at the cost of reduced water-transport efficiency, thereby improving its fitness in more arid environments [33]. In addition, wood density is closely related to the mechanical strength and hydraulic stress of plants, reflecting a trade-off between water-transport efficiency and resistance to drought-induced xylem embolism, as well as a trade-off between plant growth rate and survival ability (Table A1) [34,35]. *Acer truncatum* demonstrates greater wood density, indicative of its xylem vessels’ superior mechanical strength. This characteristic allows the vessels to resist implosion under the negative pressure that can occur during drought conditions [21,36]. Compared with the Populus species, the more negative *P*_50_ and *P*_88_ and larger hydraulic safety margin of *Acer truncatum* reflect its greater hydraulic safety in response to hydraulic imbalance induced by drought [19]. Consistent with our research findings, many studies have also shown a trade-off between hydraulic efficiency and safety. For example, a study on five poplar tree species with different growth rates revealed a close relationship between the xylem pressure causing 88% loss of hydraulic conductivity (*P*_88_) and hydraulic efficiency [37]. In addition, a study on tropical karst rainforests showed a significant positive correlation between *P*_50_ and *K*_s_, indicating that the lower the hydraulic conductivity, the stronger the resistance to cavitation [38].

The differences in pit structure of the two tree species also contributed greatly to their divergence in xylem hydraulic efficiency and safety [18,24,32]. Compared with *Acer truncatum*, the *Populus* species has a larger pit membrane area. Although this helps to enhance the permeability of water between neighboring vessels, the spreading of embolism is more likely to happen between vessels through pits [39]. Conversely, smaller pits are often linked to thicker and less permeable pit membranes, increasing their effectiveness in preventing embolism during drought stress [22,40]. The *Acer truncatum*’s smaller pit membranes and narrower pits further bolster its resistance to embolism [41]. Additionally, *Populus* species feature a lower proportion of pit openings, enhancing their ability to withstand the stretching of pit membranes under drought conditions, which in turn increases their resistance to mechanical damage and lowers the risk of embolism [40]. This study examines the structural traits of tree pits in two species, revealing a notable trade-off between hydraulic efficiency and safety at the pit level.

### 3.3. The Fast Versus Slow Growth Strategies

Different tree species have different adaptability to drought that is usually related to their potential growth rate. In accordance with their distinctly different growth rates, the two tree species under investigation exhibited significant differences in several functional characteristics associated with resource acquisition and utilization. Tree species with different growth rates may exhibit different water-use strategies. Some species support rapid growth through efficient water transport and prodigal water use, while other tree species adopt a more conservative hydraulic strategy that is associated with improved drought tolerance [42]. Species employing a fast growth strategy tend to exhibit greater sensitivity to drought conditions [43], which may stem from their need to achieve a balance between boosting drought tolerance and maintaining growth rate [44,45]. Functional trade-offs in xylem hydraulics are critical in shaping a plant’s resource-use strategy, determining how it harmonizes resource acquisition with drought tolerance. This trade-off conforms to the commonly existing fast–slow economics spectrum across species [29,46].

Our findings indicate that *Populus* species follow an acquisitive resource-use strategy, characterized by relatively low safety and high efficiency in water utilization. This approach sharply contrasts with the conservative resource-use strategy observed in *Acer* species. Meanwhile, compared with *Acer truncatum*, the higher hydraulic conductivity and carbon assimilation capacity of the *Populus* species contributed to its higher growth rate. The high *K*_l_ of the *Populus* species gives it a competitive advantage by allowing for higher leaf photosynthetic gas exchange rate and hence greater growth potential [47,48]. However, tree species with a “fast” growth strategy require greater water-transport efficiency, but also face a greater risk of embolism [49]. In addition, previous research indicates that improved water supply conditions may result in more extravagant water use, thus reducing water-use efficiency [30,47,48]. This reveals a trade-off between branch water-transport efficiency and leaf water-use efficiency. These two tree species show clear contrasts in growth rate and functional traits, reflecting their different strategies for resource acquisition and utilization. The *Populus* species adopts a “fast” strategy to quickly acquire resources and grow rapidly, while the *Acer* species chooses a “slow” strategy that contributes to more efficient resource utilization and greater resistance to environmental stress. This difference is consistent with the theory of the fast–slow economics spectrum, revealing the diverse strategies of different tree species in environmental adaptation [7,8,10,29].

## 4. Materials and Methods

### 4.1. Study Site and Plant Materials

The research was conducted at Shenyang Water Bay Park (41°45′19′′ N, 123°25′48′′ E), situated in Shenyang City, Liaoning Province, in Northeast China. The park is located near the Hun River in Shenyang, with relatively high soil moisture content and a zonal brown soil meadow soil type [50]. The frost-free period is about 150 days [51]. The park is far away from the city center, with relatively low levels of human interference. The sampling and measurements were carried out during the growing seasons of 2022 (August–September) and 2023 (July–September). The *Populus alba* × *P. berolinensis* and *Acer truncatum* trees were about 15 and 25 years old, respectively, with diameters at breast height of 22.30–29.21 and 14.23–16.52 cm, respectively. These two tree species have similar growth conditions in the park. The study area has an exhibited continental monsoon climate with precipitation concentrated in summer, cold and dry winters, and hot summers [52]. The highest and lowest mean monthly temperatures occur in January and July, at −9.0 °C and 26.2 °C, respectively. The annual average precipitation was 554.8 mm, most of which was concentrated from June to August [53]. In order to avoid the impact of the wood type different as a confounding factor for the comparative study, we selected two species both having diffuse-porous wood.

### 4.2. Tree-Ring Width and Basal Area Increment

In September 2022, we randomly selected 15 healthy trees from each species and measured the diameter at breast height (DBH) for all sampled individuals. Following this, we extracted tree cores from the selected samples utilizing a 5.15 mm diameter increment borer at the DBH height. The DBH range of *Populus alba* × *P. berolinensis* trees was 22.3 to 29.2 cm, while the DBH range of *Acer truncatum* was 14.2 to 16.5 cm. Core samples were allowed to dry naturally, and then glued, mounted on wooden staves, and polished in a sequential manner with 400, 600, and 1000 mesh sandpapers in order to obtain clearly visible tree-rings. Polished cores were scanned at 1200 dpi using a Perfection V800 scanner (Perfection V800, Epson America, Inc., Los Alamitos, CA, USA). Subsequently, the widths of the tree rings were measured with WinDENDRO 2022 software (Regent Instruments, Quebec, QC, Canada), attaining an accuracy of 0.001 mm. The COFFCHA program [54] was utilized for cross-dating, and a standard chronology based on tree-ring width was used for comprehensive statistical analysis to identify reliable periods through expressed population signals (EPSs) exceeding 0.85, thereby confirming the accuracy of tree-ring measurements. To accurately reflect the differences in radial growth characteristics between *Populus alba* × *P. berolinensis* and *Acer truncatum*, the basal area increment (BAI, cm^2^ yr^−1^) and cumulative basal area (CBA, cm^2^) at the same cambial age was calculated as follows:

(1) $${\mathrm{BAI}}_{i}=\pi \times \left(R_{i}^{2}-R_{i-1}^{2}\right)$$

where $R_{i}$ and $R_{i-1}$ represent the radius at breast height of the tree in the current and previous year, respectively;

(2) $$\mathrm{CBA}=\pi \times r_{i}^{2}$$

where $r_{i}$ represents the cumulative tree annual ring width from the first to the *i*-th year.

We calculated the BAI*_i_* at cambium ages from 1 to 12 years and CBA at cambium age of 12 years (CBA_12_) of each species.

### 4.3. Stem Hydraulic Traits

In August 2022, we collected approximately 1.5 m long samples from branches of selected tree species. These samples were immediately sealed in black plastic bags with moist paper towels and brought back to the laboratory. To ensure accurate measurements, before conducting the experiment, we cut about 10 cm from the end of each branch and soaked it in water for 1 to 2 h to restore the water content of the branches. Subsequently, unbranched stem segments measuring 25 cm in length and 1 cm in diameter were excised underwater from the branches. The stem hydraulic conductivity (*K*_h_) was determined by applying a 50 cm differential static water pressure to a degassed and filtered (0.22 μm pore size) 20 mmol L^−1^ KCl solution, which was forced through the stem segments. The liquid flow rate was then measured using a pipette over a specified time interval. This data collection was critical for accurately determining the initial hydraulic conductivity (*K*_h_), which is expressed in units of kg m^−1^ s^−1^ MPa^−1^. The calculation of *K*_h_ was performed as follows:

*K*_h_ = *J*_v_/(∆P/∆L) (3)

where *J*_v_ is flow rate of through the stem segment (kg s^−1^), ∆P is the pressure difference between the two ends of the stem (MPa), ∆L is the length of the stem segment (m).

Branches were flushed with flushing equipment for flushing. This flushing process was conducted for a duration ranging from 20 to 30 min, with the primary objective of removing any vessel embolism present in the branch. Subsequently, the maximum hydraulic conductivity (*K*_max_, kg m s^−1^ MPa^−1^) was measured.

The percentage loss of hydraulic conductivity (PLC, %) was calculated as follows:

PLC = 100 × (1 − *K*_h_/*K*_max_) (4)

After measuring the hydraulic conductivity of the stem segments, the stained cross-sectional area was measured after allowing a 0.1% solution of basic fuchsin dye to infuse in the stem segments overnight at a gravitational pressure of 5 kPa and recorded as the sapwood area (SA, mm^2^). Total leaf area at the distal end of the stem segments was quantified using a scanner (HP Scanjet G3110, Hewlett-Packard Development Co., Ltd., Beijing, China) and Image J v1.54d software (National Institutes of Health, Bethesda, MD, USA) to calculate leaf area (LA, mm^2^).

The scanned leaves were dried in an oven at 60 °C for 48 h until a constant weight was reached. Leaf mass per area (LMA, g m^−2^) was defined as the ratio of leaf dry weight to leaf area [55]. The Huber value was defined as the ratio of sapwood area to leaf area (SA/LA).

The sapwood-specific hydraulic conductivity (*K*_s_, kg m^−1^ s^−1^ MPa^−1^) was calculated as follows:

*K*_s_ = *K*_h_/SA (5)

The leaf-specific conductance (*K*_l_, ×10^−4^ kg m^−1^ s^−1^ MPa^−1^) was calculated using the following formula:

*K*_l_ = *K*_h_/LA (6)

### 4.4. Stem Vulnerability Curves and Hydraulic Safety Margin

Before the measurement, a stem section of the branch with a length of 27.4 cm was intercepted underwater. After measuring the initial hydraulic conductivity, it was fixed in a customized rotor of a high-speed centrifuge (CTK20K, Xiangyi Centrifuge Instrument Co., Ltd., Changsha, China). The branches were exerted a series of increasing higher tension in a step-wise manner (0.088, 0.3, 0.6, 0.9, 1.2, 1.5, 1.8, 2.1, 2.4, 2.7, 3, 3.5), with branches rotated at each specified tension for three minutes. After each centrifugation treatment, the segments were equilibrated underwater for three to five minutes before *K*_h_ determination.

The water potentials corresponding to 50% and 88% loss of hydraulic conductivity (*P*_50_ and *P*_88_, MPa) were obtained through a three-parameter sigmoidal model to fit the vulnerability curves. On consecutive sunny days in September selected from 12:00 and 13:00 PM, sunny, healthy twigs were cut and excised branches were immediately inserted into water-filled centrifuge tubes to minimize water loss and subsequently sent to the laboratory for measurement within one hour. The midday water potential (Ψ_md_, MPa) was quantified using a pressure chamber (PMS1000, Corvallis, OR, USA). The xylem hydraulic safety margin (HSM) is defined as the difference between Ψ_md_ and the *P*_50_ and *P*_88_ [56,57].

### 4.5. Xylem Anatomical Traits

Stem segments 2 cm long were selected from branches, stripped of bark, measured for volume by the water displacement method. The segments were then dried at 60 °C for 72 h to obtain dry weight. Wood density (WD, g cm^−3^) was calculated as the ratio of dry weight to volume. Six samples of each species were taken, debarked, and sliced into 20 µm thick slices using a sliding microtome (Model 2010-17, Shanghai Medical Instrument Company, Shanghai, China). The sections were stained with a 0.1% methylene blue solution to make temporary slides, and pictures of xylem anatomy were taken with a ×10 optical microscope (Leica ICC50, Wetzlar, Germany). Vessel diameter (*D*, μm) and vessel density (VD, no mm^−2^) were calculated with the Image J v1.54d software.

### 4.6. Pit Anatomical Traits

The stem segments used to measure the pit traits were from the same individuals as those used to measure the hydraulic traits. Stem segments measuring 1.5 cm in length with a smooth, flat surface were selected. Sections of approximately 25 μm thickness were cut along the longitudinal direction using a sliding microtome, and 5 to 8 intact sections were selected and immersed in distilled water. Subsequently, gradient dehydration was carried out for 10 min in different concentrations of ethanol solutions at 30%, 50%, 70%, 90%, and 95%. The samples were then immersed in 100% ethanol overnight. After dehydration, the samples were transferred to drying bottles and dried naturally in a ventilated area. The dried samples were fixed flatly in a sample holder and then plated at 10 mA (Leit-C, Neubauer Chemikalien, Münster, Germany) for 2 min. Samples were observed using an environmental scanning electron microscope (Quanta^TM^ 250, FEl Company, Hillsboro, OR, USA). During observation, the magnification was set to ×5000, ensuring that each sample contained at least 50 complete pits and that a minimum of 30 photographs was obtained. Single pit aperture area (*A*_ap_, μm^2^), area of the pit membrane area (*A*_m_, μm^2^), the aperture fraction (*F*_a_), and the pit aperture shape index (*AP*_f_) were calculated using Image J v1.54d software [58].

### 4.7. Leaf Photosynthetic Gas Exchange and Leaf Anatomy

The leaf photosynthetic traits net photosynthetic rate (*A*_n_, μmol m^−2^ s^−1^), transpiration rate (*T*_r_, mmol m^−2^ s^−1^), and stomatal conductance (*g*_s_, mmol m^−2^ s^−1^) were measured using a portable photosynthesis analyzer (Li-6400, Li Co Inc., Lincoln, NE, USA). A sunny day was selected in August 2023 for measurements from 9:30 to 11:30 AM. Three mature and healthy leaves were selected from each individual plant. The selected leaves were exposed to full sunlight. The branches containing the target leaves were severed using a high branch cutter. These branches were quickly immersed the end of the branch in water. The leaf chamber conditions were configured with a light intensity of 1000 μmol m^−2^ s^−1^ with a carbon dioxide concentration of 400 μmol mol^−1^. The relative humidity was maintained at 50%. The measurements were completed within one to two minutes. Instantaneous water-use efficiency (WUE_i_) was defined as the ratio of leaf net photosynthetic rate to stomatal conductance (*A*_n_/*g*_s_).

One leafy twig of each individual was cut and placed in a shaded bag filled with wet paper to retain moisture while being transported to the laboratory for anatomical analysis. The leaf transversed sections were cut using a razor blade, and temporary slides were made. Digital images were taken at ×10 magnification with an optical microscope. Thickness of spongy tissue (ST, μm), thickness of palisade tissue (PT, μm), thickness ratio of palisade to spongy tissue (PT/ST), and leaf thickness (LT, μm) were calculated using Image J v1.54d software.

### 4.8. Statistical Analysis

We first performed normality and homogeneity of variance tests on the data. Independent samples *t*-tests at the level of significance of (*p* < 0.05) were used to compare significant differences in functional traits between *Populus alba* × *P. berolinensis* and *Acer truncatum*. Pearson’s correlation analysis was employed to explore linear relationships between different functional traits and principal component analysis (PCA) was used to explain relationships between multidimensional variables.

## 5. Conclusions

This study reveals significant differences in xylem structure, photosynthesis, and hydraulic properties between poplar and *Acer*, reflecting their different resource-acquisition strategies. *Populus* has higher hydraulic conductivity, which promotes its rapid growth and competitiveness, while the *Acer* species is more resistant to drought and drought-induced xylem embolism. These hydraulic properties are closely related to their anatomical structure, reflecting a trade-off between water-transport efficiency and safety. As climate warming and drought events intensify, the selection of tree species with greater resistance to embolism and more conservative resource-utilization strategies can help mitigate the effects of drought on afforestation. This study provides important theoretical support for the selection of tree species for afforestation in water-constrained areas.

## Figures and Tables

**Table 1:** Physiological parameters related to hydraulic safety of the two studied tree species. Ψ_md_, midday leaf water potential; *P*_50_, xylem water potential corresponding to 50% loss of hydraulic conductivity; *P*_88_, xylem water potential corresponding to 88% loss of hydraulic conductivity; HSM_50_, hydraulic safety margin corresponding to 50% loss of hydraulic conductivity; HSM_88_, hydraulic safety margin corresponding to 88% loss of hydraulic conductivity. Different lowercase letters in the same column signify that the index is significantly different between the two tree species (*t* test, *p* < 0.05). Values are means ± 1 SE (*n* = 4).

Tree Species	Ψ_md_ (MPa)	*P*_50_ (MPa)	*P*_88_ (MPa)	HSM_50_ (MPa)	HSM_88_ (MPa)
*Populus alba* L. × *P. berolinensis* Dippel	−1.72 ± 0.21 b	−1.26 ± 0.12 a	−1.61 ± 0.17 a	−0.98 ± 0.14 b	−1.26 ± 0.12 b
*Acer truncatum*	−1.02 ± 0.18 a	−2.50 ± 0.23 a	−3.12 ± 0.12 b	0.31 ± 0.27 a	1.82 ± 0.14 a

**Table 2:** Leaf morphological and structural characteristics of the two studied tree species. LS, leaf size; LMA, leaf mass per area; LT, leaf thickness; PT, thickness of palisade tissue; ST, thickness of sponge tissue; PT/ST, palisade to spongy tissue thickness ratio. Different lowercase letters in the same column signify that the index is significantly different between the two tree species (*t* test, *p* < 0.05). Values are means ± 1 SE (*n* = 6).

Tree Species	LS (cm^2^)	LMA (g m^−2^)	LT (μm)	PT (μm)	ST (μm)	PT/ST
*Populus alba* × *P. berolinensis*	10.60 ± 0.8 b	108.58 ± 8.9 a	172.20 ± 2.7 a	80.72 ± 1.4 a	53.29 ± 1.2 a	1.53 ± 0.02 a
*Acer truncatum*	29.20 ± 2.0 a	48.23 ± 4.7 b	121.24 ± 3.0 b	36.92 ± 1.6 b	44.70 ± 1.0 b	0.84 ± 0.02 b

## Data Availability

The original contributions presented in this study are included in the article. Further inquiries can be directed to the corresponding author.

## Appendix A

**Table A1:** Pearson correlation coefficients among different functional traits of the two tree species.

	LA	LMA	WD	SA	WA	*K*_l_	*K*_s_	Hv	*A*_n_	*g*_s_	*T*_r_	WUE_i_	*A*_ap_	*A*_m_	*F*_a_	*A*_pf_	*D*	VD	*P*_50_	*P*_88_
LA																				
LMA	−0.78 **																			
WD	0.61 *	−0.75 **																		
SA	0.12	0.12	0.003																	
WA	−0.38	0.64 *	−0.63 *	0.58 *																
*K*_l_	−0.64 *	0.85 **	−0.83 **	0.02	0.70 **															
*K*_s_	−0.45	0.50	−0.57 *	−0.62*	0.19	0.64 *														
Hv	−0.39	0.59 *	−0.45	0.78**	0.79 **	0.56 *	−0.20													
*A*_n_	−0.70 **	0.81 **	−0.76 **	0.04	0.74 **	0.85 **	0.59 *	0.45												
*g*_s_	−0.80 **	0.82 **	−0.86 **	−0.21	0.56 *	0.92 **	0.77 **	0.27	0.90 **											
*T*_r_	−0.33	0.46	−0.54	0.16	0.67 *	0.58 *	0.20	0.38	0.72 **	0.67 *										
WUE_i_	0.53	−0.46	0.59 *	0.39	−0.09	−0.58 *	−0.63 *	0.05	−0.28	−0.65 *	−0.17									
*A*_ap_	0.26	−0.05	0.20	0.36	0.20	0.06	−0.36	0.29	0.16	0.03	0.50	0.14								
*A*_m_	−0.45	0.67*	−0.77 **	0.03	0.65 *	0.81 **	0.51	0.44	0.81 **	0.84 **	0.74 **	−0.53	0.08							
*F*_a_	−0.15	0.86 **	−0.65 *	−0.12	0.50	0.70 **	0.65 *	0.26	0.80 **	0.86 **	0.43	−0.56 *	−0.14	0.70 **						
*A*_pf_	0.57 *	−0.71 **	0.74 **	0.18	−0.47	−0.69 **	−0.68 **	−0.23	−0.64 *	−0.76 **	−0.36	0.61 *	0.52	−0.77 **	−0.70 **					
*D*	−0.73 **	0.68 *	−0.72 **	−0.32	0.52	0.78 **	0.80 **	0.14	0.88 **	0.88 **	0.58	−0.39	−0.04	0.73 **	0.88 **	−0.72 **				
VD	0.64 *	−0.59 *	0.74 **	0.19	−0.58 *	−0.72 **	−0.65 *	−0.21	−0.82 **	−0.77 **	−0.55	0.21	0.05	−0.60 *	−0.76 **	0.59 *	−0.93 **			
*P*_50_	0.27	−0.22	−0.05	0.39	0.16	−0.09	−0.20	0.11	−0.16	−0.13	0.01	−0.06	0.52	0.06	−0.22	0.30	−0.34	0.35		
*P*_88_	0.89 **	−0.74 *	0.64	0.35	−0.25	−0.81 **	−0.79 *	−0.20	−0.65	−0.86 **	0.08	0.64	0.57	−0.64	−0.84 **	0.81 **	−0.81 **	0.63	0.42	

LA, leaf area; LMA, leaf mass per area; WD, wood density; WA, total wood area; SA, sapwood area; *K*_s_, sapwood hydraulic conductivity; *K*_l_, leaf-specific conductivity; Hv, Huber value; *D*, vessel diameter; VD, vessel density; *A*_n_, leaf net photosynthetic assimilation rate; *g*_s_, stomatal conductance; *T*_r_, sap flow transpiration rate; WUE_i_, instantaneous water-use efficiency; *A*_ap_, pit aperture area; *A*_m_, pit membrane area; *F*_a_, aperture fraction; *AP*_f_, pit aperture shape index; *P*_50_, xylem water potential corresponding to 50% loss of hydraulic conductivity; *P*_88_, xylem water potential corresponding to 88% loss of hydraulic conductivity. Asterisks indicate that there is a significant correlation between the two traits (*, *p* < 0.05; **, *p* < 0.01).
